# Excessive substance use in bipolar disorder is associated with impaired functioning rather than clinical characteristics, a descriptive study

**DOI:** 10.1186/1471-244X-10-9

**Published:** 2010-01-27

**Authors:** Trine V Lagerberg, Ole A Andreassen, Petter A Ringen, Akiah O Berg, Sara Larsson, Ingrid Agartz, Kjetil Sundet, Ingrid Melle

**Affiliations:** 1 Section for Psychosis Research, Oslo University Hospital, Bygg 49, Kirkevn. 166, N-0407 Oslo, Norway; 2 Institute of Psychology, University of Oslo, Box 1094, Blindern, N-0317 Oslo, Norway; 3 Institute of Psychiatry, University of Oslo, Box 1130 Blindern, N-0318 Oslo, Norway; 4 Department of Research and Development, Diakonhjemmet Hospital, Box 23, N-0319 Oslo, Norway

## Abstract

**Background:**

There is a strong association between bipolar disorder (BD) and substance use disorder (SUD). The clinical and functional correlates of SUD in BD are still unclear and little is known about the role of excessive substance use that does not meet SUD criteria. Thus, the aims of the current study were to investigate lifetime rates of illicit substance use in BD relative to the normal population and if there are differences in clinical and functional features between BD patients with and without excessive substance use.

**Methods:**

125 consecutively recruited BD in- and outpatients from the Oslo University Hospitals and 327 persons randomly drawn from the population in Oslo, Norway participated. Clinical and functional variables were assessed. Excessive substance use was defined as DSM-IV SUD and/or excessive use according to predefined criteria.

**Results:**

The rate of lifetime illicit substance use was significantly higher among patients compared to the reference population (OR = 3.03, CI = 1.9-4.8, p < .001). Patients with excessive substance use (45% of total) had poorer educational level, occupational status, GAF-scores and medication compliance, with a trend towards higher suicidality rates, compared to patients without. There were no significant group differences in current symptom levels or disease course between groups.

**Conclusion:**

The percentage of patients with BD that had tried illicit substances was significantly higher than in the normal population. BD patients with excessive substance use clearly had impaired functioning, but not a worse course of illness compared to patients without excessive substance use. An assessment of substance use beyond SUD criteria in BD is clinically relevant.

## Background

Comorbid bipolar disorder (BD) and substance use disorder (SUD) have been found to be highly prevalent in both epidemiological and clinical studies, with rates of SUD in subjects with BD ranging from 35-60% [1-6]. The high prevalence is found across different age groups and also in first episode BD samples [7,8].

So far, most studies in BD have investigated only substance use fulfilling SUD criteria. Investigating a broader range of substance use in BD could be relevant because people with severe mental disorders are more likely to experience negative consequences from using relatively small amounts of psychoactive substances [9]. Moderate alcohol consumption in BD is associated with more severe manic symptoms compared to abstinence, and to poorer social and familial adjustment and increased health-care use [10]. To the best of our knowledge, only one study assessed substance use in BD more globally, reporting that 46% had SUDs and 8% had SUD-subthreshold substance use. In addition, the authors indicated that another substantial proportion used illicit substances occasionally [11].

Clarifying whether there is an increased *use *of substances in BD may increase our understanding of the psychopathology underlying the increased risk of abuse or dependence. Although most studies show a large prevalence of BD and SUD comorbidity, the rates vary widely. This variation could be mirroring differences in substance use in the general population where the BD sample is recruited. In a smaller sample from an earlier part of our ongoing study, we showed elevated rates of lifetime use of illicit substances among patients with psychotic disorders (including BD) compared to the general population [12], and differences in patterns of substance use between schizophrenia and BD [13]. Due to the small number of patients with BD included in our earlier report, a separate comparison of BD patients with the general population sample was not implementable. Thus, there is a need for studies comparing BD subjects with reference populations on substance use and they should be done with samples from the same geographical area within the same time period.

In the current literature, BD with comorbid SUD is consistently referred to as associated with a poorer disease course and with reduced functioning compared to BD without SUD. The findings regarding the effects of SUD on BD are however divergent. To explore this more thoroughly we did a search in PubMed (terms bipolar disorder, substance abuse and outcome), and in addition tracked all cited references in key publications (Additional file 1). The main finding from this search was that the only consistently reported findings were delayed recovery and lower remission rates [14-22] as well as faster relapses [14,23-25] in groups of BD patients with SUD (both lifetime/current substance - and/or alcohol use disorders) compared to BD without SUD. Furthermore, there appears to be extensive evidence for elevated suicidality rates in BD with SUD compared to BD without [18,20,26-37], although several studies also report no significant differences [19,38-42]. Medication compliance rates are also relatively consistently reported to be lower in BD with SUD compared to BD without [18,19,29,43-46] although a few studies report lack of differences [38,42]. Another consistent finding is that the prevalence of psychotic symptoms does *not *appear to be elevated among BD patients with SUD compared to patients without [18,19,28,38,47,48], and there is neither a tendency towards increased numbers of affective episodes [19,27,31,48,49].

The findings are more divergent regarding rapid cycling; as some studies did [38,40,50-52] and some did not [19,29,53] find this to be more prevalent in the SUD patients. The same inconsistency is found for the prevalence of mixed episodes, some studies found this phenomenon to be more common [14,18,39,50,54] while others did not [17,47,55] in the SUD patients. There are also inconsistencies regarding age of onset for BD; here some report earlier onset for patients with SUD [26,29-31,50,51,56,57] while others do not find any differences compared to BD patients without SUD [18,19,38,47,55,58]. Studies also diverge as to whether affective symptoms are of increased severity in BD patients with SUD compared to BD patients without [18,21,26,39,42,47,49,50,59,60]. Furthermore, the number of hospitalizations or days in hospital is found to be elevated in BD patients with SUD in some studies [29,31,50,55,61-64] as opposed to in others [18,26-28,38,48,56,65].

Findings concerning other functional variables such as decreased global functioning [19,26,38,39,47,48,56,60,66], social functioning [20,21,27,29,38,58,60,67], educational level [19,20,26,31,38,50,56,60], and quality of life [20,21,26,58,60,61] in BD with SUD also diverge. Finally, some studies find lower employment status in BD with SUD compared to BD without [21,24,29,67] while others do not [28,43,50,56], and two studies even find better employment rates in BD with SUD [19,61]. The current evidence therefore suggests that BD with comorbid SUD is clearly associated with worsening of *some *clinical and functional characteristics: Length of affective episodes and relapse rates, risks of suicidality and compliance to medication. However, substance abuse does not appear to be as consistently associated with a more severe course and outcome as frequently indicated in the literature.

In the present study, we aim at investigating differences in relevant outcome variables in a sample of BD patients with and without substance use. The present paper is based on a cross-sectional study of consecutively referred patients with BD from a catchment-area based psychiatric service, and a population survey of the use of illicit substances in the same area within the same time period. Our aims were to answer the following questions:

1) Is the rate of lifetime use of illicit substances higher in the patient sample than in the reference population?

2) Do patients with and without excessive substance use, defined as SUD and/or excessive use, differ on clinical and functional characteristics, in terms of disease course variables, current symptom levels and functioning?

## Methods

### Participants

125 patients with DSM-IV bipolar disorder (BD I n = 71 and BD II n = 54), participated in the study. The sample is part of an ongoing study of schizophrenia and bipolar disorder (the Thematically Organized Psychosis Research - TOP study). The BD patients were consecutively recruited between 2003 and 2007 from the psychiatric units (in- and outpatient) of the three major hospitals in Oslo. The exclusion criteria for all participants were: history of moderate/severe head injury, neurological disorder, mental retardation, age outside the range of 18-65 years, and not speaking a Scandinavian language. All participants gave informed consent, and the project was approved by the Regional Committee for Medical Research Ethics and the Norwegian Data Inspectorate.

A sample from the general population was used as a reference group for rates of lifetime use of illicit substances, collected by the Norwegian Institute for Alcohol and Drug Research (SIRUS). SIRUS regularly conducts surveys of the Norwegian population's consumption of illicit substances by personal interviews via standardized questionnaires. Subjects are randomly selected according to a detailed selection protocol and weighted to age, gender and address [68]. For the purpose of this study, we used a reference group of 327 subjects from 2004 SIRUS data for Oslo, with participants aged 18-65. There was no age difference between the patient group and the reference group (35.6, SD 11.7 vs. 36.0, SD 12.0), but the proportion of women was significantly greater in the patient sample (64.8% vs. 51.4%, Χ^2 ^= 6.59, df = 1, p = 0.010).

### Clinical assessment

Clinical assessment was carried out by trained clinical psychologists and psychiatrists. Diagnoses were established using the Structured Clinical Interview for DSM-IV, modules A-E [69]. General non-psychotic symptoms were assessed by the Positive and Negative Syndrome Scale (PANSS) [70], depressive symptoms with the IDS-C [71], (hypo)manic symptoms with the Young Mania Rating Scale (YMRS) [72] and current functioning by the Global Assessment of Functioning Scale (GAF) [73], split version [74]. The Medication Adherence Rating Scale (MARS) [75] was used to measure compliance to medication. A total of 103 patients (82.4%) completed the MARS. Eight patients (6.4%) did not complete because they were not using any medication at the time of the evaluation. Among the patients not completing the MARS, there was no significant difference in the proportion with or without excessive substance use.

All interviewers were trained based on the training program at UCLA (CA, USA) and participated in regular diagnostic consensus meetings. A good inter-rater reliability was achieved with an overall kappa score of 0.77 (95% CI: 0.60-0.94). The reliability for symptom assessments was also good, with an intraclass correlation coefficient (1.1) of 0.71 for the PANSS general subscale, and of 0.86 for both symptom and function GAF scores (for details, see Ringen et al. 2007b).

Some of the variables frequently reported in the literature, like prevalence of mixed episodes and rapid cycling, were not investigated in the present study, due to a study design that did not focus on specific characteristics of the affective episodes. Disease course was assessed by means of SCID criteria, which lack the specificity needed for satisfactory reliability of such phenomena.

### Substance use assessments and excessive substance use definitions

Patients were asked for age at first experience with drinking alcohol and using non-alcoholic drugs (including non-prescribed anxiolytic and hypnotic medicines). Lifetime use of all substances through age intervals (age 12-15, 16-20, 21-27, 28-44, 45-60, 60+) was registered separately in categories of daily, weekly, monthly or occasional/no use within each interval, based on the possibility of different use patterns and of differences in the pathophysiological influence of substances across different age periods. Predominantly *daily *use of alcohol and predominantly *weekly *use of a non-alcoholic substance throughout an age interval across a minimum of 4 years were considered *excessive*, and substance use according to these definitions is subsequently termed *excessive use*. Structured interviews about substance use during the past 6 months were performed. Alcohol use was assessed by number of units and non-alcoholic substance use by number of incidents. Different non-alcoholic substances were asked for specifically and the use was quantified by totaling the number of incidents recalled. Urine samples were also collected and corresponded well with patients' own reports of consumption of non-alcoholic substances in previous weeks [13]. There were no statistically significant differences among the levels of substance use (number of units of alcohol or number of incidences of use of non-alcoholic substances) the last 6 months between patients fulfilling SUD criteria and patients with excessive use. But these two groups combined differed significantly from the patients with neither SUD nor excessive substance use. Thus, for the subsequent analyses, patients with SUD and patients with excessive use were aggregated in an "*excessive substance use group*". Patients with none of these are subsequently named "*no use group*".

The mean age was 34.8 (SD 11.8) in the excessive substance use group and 36.2 (SD 11.2) in the no use group (n.s.). In the excessive substance use group, 54% were female, which was significantly different from the no use group, where 74% were female (Χ^2 ^= 5.608, p = 0.018). 93% were Caucasian in the excessive substance use group, and 90% in the no use group (n.s.). Median duration of illness was 9.5 years (IQR 12) in the excessive substance use group and 11.5 years (IQR 16.75) in the no use group (n.s.).

### Statistical procedure

All analyses were done using the Statistical Package for the Social Sciences (SPSS) version 16.0. The limit for significance was set to 0.05 (two-sided). Chi-square tests and Fisher's exact tests were used when investigating group differences on categorical data. Group differences in independent samples were explored with Student's t-tests and ANOVAs on normally distributed continuous variables and Mann Whitney U-tests and Kruskal Wallis tests for variables with a skewed distribution. The distribution of skewed variables is presented through medians and interquartile ranges (IQR). Binary logistic regression analysis was used for calculating odds ratios, controlling for relevant variables. Correlations between group membership, outcome measures and background variables that might mediate their relationships were explored through Pearson and Spearman rank correlations. The presence of possible confounder variables was explored through hierarchical multiple regression analysis. The Kaplan-Meier Survival Analysis was used to compare time in remission across the two groups.

For the analyses related to research question 2, the levels of relevant demographic and clinical measures were compared for the excessive substance use group versus the no use group. Since the distribution of sex was significantly different in the excessive substance use group and the no use group, it was considered a potential confounder in the associations between group membership and outcome variables and possible mediating effects were investigated.

## Results

The prevalence of lifetime use of illicit substances was 65% in the patient sample and 40% in the general population sample. When corrected for age and sex, the risk of lifetime use of illicit substances was significantly and three times greater in the patient sample compared to the reference population (OR = 3.03, CI = 1.9-4.8, p < .001).

The prevalence of SUDs and excessive substance use are presented in Table 1.

**Table 1:** Prevalence of lifetime substance use disorders and of excessive use in patient sample, N (%)

	N = 125
SUD total	38 (30.4)
Alcohol use disorder	26 (20.8)
Cannabis use disorder	15 (12.0)
Other non-alc. substance use disorder	14 (11.0)
Excessive use total	18 (14.4)
Excessive alcohol use	7 (5.6)
Excessive cannabis use	13 (10.4)
Excessive use other non-alc. substances	2 (1.6)
SUD + excessive use	56 (44.8)

SUD and excessive substance use are here mutually exclusive categories. Within these categories, some patients meet the criteria for two or more substance use disorders or can excessively use two or more substances.

Regarding clinical and functional outcome variables (Table 2), we found that the no use group had significantly more years of education than the patients with excessive substance use (15.1, SD 2.9 versus 13.5, SD 2.6, p = 0.001). The proportion that was employed/full time students was significantly smaller in the excessive substance use group (21% versus 45%, p = 0.006). We also found that the excessive substance use group had significantly lower mean GAF S and F scores than the no use group (52.9, SD 10.7 versus 59.7, SD 11.1, p = 0.001 and 50.3, SD 11.3 versus 57.2, SD 12.1, p = 0.002, respectively). Correlation analyses revealed that number of years of education correlated with the excessive substance use group (Pearson's r = -0.29, p = 0.001), and GAF S and F scores (GAF S: Pearson's r = 0.22, p = 0.016, GAF F: Pearson's r = 0.21, p = 0.018). After correction for number of years of education, age and sex, there was still a significant association between excessive substance use and lower GAF S score (group membership entered as last variable, β = -0.24, p = 0.009), and lower GAF F score (β = -0.20, p = 0.034). Furthermore, the excessive substance use group had a significantly higher median MARS score, i.e. was less compliant by self-report than the no use group (8, IQR 5 versus 7, IQR 3, p = 0.010). There was also a strong trend that the excessive substance use group had more suicide attempts than the no use group (p = 0.053).

**Table 2:** Clinical course and functional outcome variables in the "excessive substance use" group versus the "no use" groups

	Excessive substance	No use	Test statistics/p-value	Effect sizes
	use group, N = 56	group, N = 69		
IDS-C, median (IQR)	16.5 (17)	13.5 (20)	U = 1640.5, p = 0.853^a^	
YMRS, median (IQR)	2 (3)	2 (5)	U = 1730.5, p = 0.393^a^	
PANSS general, mean (SD)	26.1 (5.9)	24.6 (6.0)	t = -1.384, df = 122, p = 0.169^d^	
Age at onset of BD (years), median (IQR)	20 (9)	19 (10)	U = 1894.0, p = 0.962^a^	
Duration of illness, median (IQR)	9.5 (12)	11.5 (16.75)	U = 1739.0, p = 0.407^a^	
In remission, n (%)	19 (35)	31 (46)	X^2 ^= 1.515, p = 0.218^b^	
Time in remission, months, median (IQR)	3 (4)	5 (7.25)	X^2^ = 2.511, p = 0.113^c^	
No. of elevated mood episodes, median (IQR)	3 (8.5)	2 (4)	U = 1619.0, p = 0.288^a^	
No. of depressive episodes, median (IQR)	4 (9)	3 (8)	U = 1716.0, p = 0.604^a^	
Bipolar disorder type, BD I, n (%)	30 (54)	41 (59)	X^2 ^= 0.431, p = 0.512^b^	
Psychosis, n (%)	20 (36)	32 (48)	X^2 ^= 1.604, p = 0.205^b^	
No. of suicide attempts, median (IQR)	0 (1)	0 (1)	U = 600.0, p = 0.053^a^	
Hospitalized (lifetime), n (%)	35 (65)	45 (67)	X^2 ^= 0.074, p = 0.786^b^	
No. of admissions, median (IQR)	1 (2.8)	1 (3)	U = 1814.0, p = 0.745^a^	
Duration of admissions (months), median (IQR)	1.5 (4.2)	3.3 (5)	U = 568.0, p = 0.056^a^	
MARS score, median (IQR)	8 (5)	7 (3)	U = 915.5, **p = 0.010^a^**	Diff. in mean rank = 15.17
Years of education, mean (SD)	13.5 (2.6)	15.1 (2.9)	t = 3.307, df = 123, **p = 0.001^d^**	Cohen's d = 0.596
Currently employed/full time students, n (%)	12 (21)	31 (45)	X^2 ^= 7.564, **p = 0.006^b^**	Phi = -0.246
Marital status (married/living as married), n (%)	20 (36)	26 (38)	X^2 ^= 0.051, p = 0.821	
GAF S, mean (SD)	52.9 (10.7)	59.7 (11.1)	t = 3.458, df = 123, **p = 0.001^d^**	Cohen's d = 0.624
GAF F, mean (SD)	50.3 (11.3)	57.2 (12.1)	t = 3.112, df = 123, **p = 0.002^d^**	Cohen's d = 0.561

IQR = interquartile range. ^a^Mann Whitney U-test, ^b^Chi-square test, ^c^Log rank (Mantel Cox) test, ^d^Student's t-test.

We found no significant differences in affective and general symptomatology as measured by the IDS-score, YMRS-score or PANSS general score between the patients with and without excessive substance use (Table 2). The proportion of patients in remission was not significantly different across the two groups, nor was time in remission. No significant differences were found between the groups in age at onset of BD, number of elevated episodes (manic/hypomanic), number of depressive episodes, or bipolar subtype distribution (BD I vs. BD II). No significant differences between the groups were found in lifetime prevalence of psychotic symptoms. Regarding the latter, a separate analysis comparing patients that only excessively used psychoactive substances known to induce psychotic symptoms (cannabis and centrally stimulating agents) with the no use group revealed no significant differences (Χ^2 ^= 0.059, p = 0.564). No significant differences were found regarding lifetime hospital admission or total number of admissions. Among the ones admitted, there was a trend that the duration of admissions was shorter in the excessive substance use group (p = 0.056). These analyses were repeated for only patients fulfilling SUD criteria versus the no use group (excessive use patients excluded), revealing no additional significant differences between the two groups. To investigate whether alcoholic and non-alcoholic substances influenced outcome in different directions, the alcoholic and non-alcoholic excessive substance use groups were compared with the no use group separately. This yielded no new significant associations.

## Discussion

The main findings of the present study are that patients with BD had a significant increase (OR of 3) of lifetime use of illicit substances compared to the general population, and that excessive substance use was associated with poorer functioning but not with worse illness course characteristics or current symptom levels.

To the best of our knowledge, this is the first study to report lifetime illicit substance use in a clinical sample of BD patients compared to the reference population. Our data indicate that the risk is greater than in the general population not only to develop SUDs, but also to use such drugs at a SUD-subthreshold level. Despite large research efforts, the mechanisms involved in the increased substance use in BD are not known. Several studies have found increased impulsivity and novelty seeking in BD patients [76,77], which have also been linked to substance use [78,79]. This could partially explain the increased tendency to experiment with and excessively use substances among subjects with BD [80]. The same could be true for Behavioral Approach System (BAS) dysregulation, in which high BAS sensitivity has been linked to both increased risk of (hypo)manic episodes [81] and substance abuse [82]. Searching for potential protecting factors in BD subjects not developing SUD could be a worthwhile approach for future studies.

The total alcohol use disorder rate of 21% found in the present study was in the lower range of earlier clinical reports on samples consisting of both BD I and II disorders [20,30,83], and the higher SUD rates in males compared to females is in accordance with earlier findings [57,58]. Thus, the somewhat higher proportion of females in our sample could explain the lower alcohol use disorder rate. Furthermore, both drug use and alcohol use patterns differ between countries and cultures. The average alcohol intake in Norway is significantly lower than the European continent, the UK and the US [84,85], which could also explain the lower risk of alcohol use disorder in the patient group in the present study.

There were several indicators of a poorer functioning in the excessive substance use group compared to the no use group, including length of education and employment rate. The hierarchical multiple regression analyses also indicated direct associations between excessive substance use and lower GAF scores that were not mediated by years of education. Although earlier studies are inconsistent, our findings of poorer functioning in the excessive substance use group are in line with several studies showing greater functional impairment associated with comorbid SUD [20,21,29,60]. The excessive substance use group also had poorer compliance, which is in accordance with earlier research [45,46]. The trend towards shorter hospital admissions found in the excessive substance use group could also be interpreted as reduced compliance, as shorter admissions may be an expression of treatment non-compliance. Alternatively, inpatient treatment facilities are not optimal for treating BD patients with excessive substance use which may lead to shorter inpatient treatment. Shorter durations of psychiatric hospital admissions among patients with comorbid mental illness and SUD have also been found in earlier studies [86].

We did not find evidence that the presence of excessive substance use was associated with more severe BD specific disease characteristics. Earlier studies mainly investigated DSM-IV SUD, which is more narrowly defined than the present study's excessive substance use category. However, when we analyzed the narrowly defined SUD group, we did not find different results compared to the excessive substance use group. Furthermore, we found that the substance use levels among patients with excessive use were similar to patients with SUDs. Comparing our results with studies investigating SUD should therefore be relevant. The present lack of association between excessive substance use and current affective symptomatology is in line with several other studies finding no differences across groups defined by SUD in these variables [18,50]. It has also been hypothesized that SUD may trigger BD in individuals without a great constitutional vulnerability for the disorder [48,87]. Thus, a lack of worsening of BD illness characteristics in the presence of SUD may be explained by a lower vulnerability. Our finding of no relationship between excessive substance use and an earlier onset of the BD is consistent with some studies [55] but in contrast to others [29], and these discrepancies are difficult to explain. The present lack of significant differences in remission variables was unexpected, since prolonged affective episodes are found quite consistently by earlier research [20]. However, there were numerical differences between the groups in the expected direction on these variables, so this difference could reach statistical significance in a larger sample. Furthermore, the present finding of no relationship between excessive substance use and number of affective episodes is in line with previous research [49] although this is sparsely investigated. Finally, the similar distribution of bipolar subtypes across the groups in our study converges with some studies [20,83], but is contrary to those finding higher SUD rates in bipolar I disorder compared to bipolar II disorder [1,6]. Our findings of no differences in BD illness severity between patients with or without excessive substance use is in accordance with a recent study on BD I disorder with or without SUD on several proxies for BD severity [27].

The trend towards increased suicidality rates as well as the lower GAF S scores found in the excessive substance use group in the present study, could be signs of a poorer general psychiatric outcome not linked to a more severe BD. Increased suicidality is seen in a number of psychiatric disorders and has been found associated with SUD alone [88], and with the combination of SUD and a variety of psychiatric disorders [89-91]. Thus it appears reasonable to link the increased suicidality more to the excessive substance use per se than to a more severe BD course. The lower GAF S scores in our excessive substance use group were not reflected in increased symptoms as measured by the IDS and the YMRS as could be expected, and may also be directly related to the substance use itself or to the burden of having two disorders. In summary, excessive substance use does not appear to be related to more severe specific BD illness characteristics, but to a more severe general psychiatric outcome in terms of worse global clinical features unspecific to psychiatric diagnosis and frequently seen in association with substance abuse alone.

Our finding concerning psychosis is in accordance with previous studies reporting a lack of association between SUD and higher lifetime rates of psychosis in BD [18]. This is not surprising given that these studies did not specifically investigate the use of cannabis and centrally stimulating agents known to induce psychotic symptoms during intoxication [92,93] and increase the risk of psychotic disorders [94,95]. The lack of association between psychosis and excessive use of these psychosis inducing substances found in the present study is somewhat surprising, but could be related to a high psychosis frequency in general in BD patients, thereby reducing the relative effect of substance use.

The present study's approach of adding patients with a SUD-subthreshold excessive substance use to the SUD group has additional value, in that we demonstrate that SUD criteria are not necessarily the appropriate cut-off when addressing and assessing harmful substance use in BD. Our findings may also have important implications for treatment of BD patients with excessive substance use. Because of the increased functional impairment and treatment non-compliance associated with excessive substance use, substance use should be targeted in treatment before the clinical signs of abuse or dependence have developed. Our findings further demonstrate that patients with a considerable amount and frequency of substance use may not necessarily fulfill SUD diagnostic criteria.

The inconsistency revealed in the literature regarding differences in clinical and functional characteristics between BD with and without SUD is somewhat unexpected, as several papers including reviews of the topic generally state that there is consistent evidence that a comorbid SUD is associated with more severe features. This is a relatively new field, thus citation and publication biases may be a problem. Studies also vary to a great extent in operationalizations and methodology, which may explain some of the discrepancies. Furthermore, studies setting out to answer questions about the associations between comorbid SUD and outcome in BD patients are few compared to studies that focus on other issues and report relationships between comorbid SUD and outcome as secondary findings. Also, since only a few studies display effect sizes in addition to significance levels, little is known about the strength of the associations. Thus, there is a great need for more well-designed and hypothesis-driven studies addressing this question as well as future efforts to agree on methodology.

The present study has some limitations. The sample in the present study was too small to investigate current use levels or non-alcoholic substance types separately. Furthermore, since this is a cross-sectional study, no conclusions of causality may be drawn regarding the association between excessive substance use and the functional level. Thus, whether these relationships are due to negative effects from the excessive substance use, or related socioeconomic factors, cannot be determined. Also, the sample size is relatively small, with an increased risk for type II errors. However, there are few substantial numerical differences between the groups, thus an increase in sample size would not lead to additional significant differences. This is a well characterized catchment area study, covering both in- and outpatient units including substance abuse clinics.

## Conclusions

The current findings show that there is a significant increase in illicit substance use in BD compared to general population with an OR of 3. Patients with excessive substance use have indications of impaired functioning and some signs of a more severe general psychiatric outcome, but not worse illness course characteristics or current symptom levels. This has implications for current treatment and should lead to more research into the underlying psychopathological mechanisms.

## Competing interests

The authors declare that they have no competing interests.

## Authors' contributions

TVL participated in planning of the current study, the collection of data, did the statistical analyses and wrote the first draft of the paper and coordinated the writing process. OAA, KS and IM participated in planning of the study, supervised the data collection and statistical analyses. PAR, AOB, SL and IA participated in the data collection. All authors have made substantial contributions to writing of the manuscript and have approved the final version.

## Pre-publication history

The pre-publication history for this paper can be accessed here:

http://www.biomedcentral.com/1471-244X/10/9/prepub

## Supplementary Material

Additional file 1 **Additional file**1**provides an overview table of the literature reported in the background section concerning the effect of substance use disorders on BD**. The table is organized in two parts; Part I: "Reported effects of substance use disorders on measures of functioning and general psychopathology", and Part II: "Reported effects of substance use disorders on measures of illness course and clinical characteristics specific to bipolar disorder". Some of the reviewed studies appear in both Part I and Part II. Click here for file
