# Supplementary material for: Excessive substance use in bipolar disorder is associated with impaired functioning rather than clinical characteristics, a descriptive study
Source: BMC Psychiatry. 2010 Jan 27;10:9. doi: 10.1186/1471-244X-10-9 (PMC2824653; doi:10.1186/1471-244X-10-9)
Supplement: Additional file 1 — Additional file1provides an overview table of the literature reported in the background section concerning the effect of substance use disorders on BD. The table is organized in two parts; Part I: "Reported effects of substance use disorders on measures of functioning and general psychopathology", and Part II: "Reported effects of substance use disorders on measures of illness course and clinical characteristics specific to bipolar disorder". Some of the reviewed studies appear in both Part I and Part II. [file 1471-244X-10-9-S1.DOC]

**Additional file 1.**

***Part I: Reported effects of substance use disorders on measures of functioning*** and general psychopathology.

| **Publication** | **Study** | **Methods and sample charcteristics** | **Type and measure of SUD** | **Type of functional outcome and general psychopathology** | | | | | | | |
| --- | --- | --- | --- | --- | --- | --- | --- | --- | --- | --- | --- |
|  |  |  |  | **Educational level** | **Employment status** | **Social functioning** | **Quality of life** | **Medication compliance** | **Global functioning** | **Suicidality** | **Hospitalization** |
| **Reich & Himmelhoch, 1974** | Lithium Clinic, Yale-New Haven Hospital, USA | N=65, BD I | Excessive alcohol use |  |  |  |  |  |  |  | Increased (level of significance not presented) |
| **Morrison et al., 1974** | VA Hospital, San Diego, California, USA | N=38, BD I | Alcoholism | No effect |  |  |  |  |  | Increased (level of significance not presented) | Increased (level of significance not presented) |
| **Winokur et al., 1995** | NIMH Collaborative Study on the psychobiology of depression, USA | N=231, BD I+II and schizoaffective disorder, 5 year prospective | AUD |  |  |  |  |  | No effect | Increased in females only | No effect |
| **Tohen et al., 1990** | McLean Hospital, Massachusettes, USA. | N=75, BD I, 4 year prospective | Alcoholism |  | Reduced, OR=8.2 (1.2-55.7) |  |  |  |  |  |  |
| **Brady et al., 1991** | VA Medical Center, Charleston, South Carolina, USA | N=20 (total N=100), BD I inpatients, 97% males | SUD |  |  |  |  |  |  |  | Increased (level of significance not presented) |
| **O’Connell et al., 1991** | St. Vincent’s Hosp. and medical center, New York, USA | N=248, BD I+II, 1 year prospective | SUD |  |  |  |  |  | Reduced |  |  |
| **Sonne et al. 1994** | Medical U. of South Carolina, USA | N=44, BD I+II, in- and outpatients | SUD current/  lifetime | Reduced | No effect |  |  |  |  |  | Increased in current SUD only |
| **Tsai et al., 1997** | Taiwan (Chinese patients) | N=158 BD I+II | AUD | No effect |  | No effect |  | No effect | No effect | No effect | No effect |
| **Bauer et al., 1997** | Providence VA medical center, Rhode Island, USA | N=103, BD I+II | SUD |  |  |  |  |  |  |  | No effect |
| **Keck et al., 1998** | U. of Cincinnati Hospital, USA. | N=134, BD I, inpatients, 1 year prospective | SUD |  | No effect |  |  | Reduced |  |  |  |
| **Pini et al., 1999** | Pisa Center, Italy | N=125, psychotic BD, inpatients | SUD | No effect | No effect |  |  |  | No effect |  | No effect |
| **Goldberg et al., 1999** | New York Hospital, USA | N=204, BD I, inpatients | SUD |  |  |  |  | Reduced |  | Increased | No effect |
| **Kusznir et al., 2000** | CAMH, Bipolar Clinic,, U. of Toronto, Canada | N=87, BD I+II, outpatients | Alcohol abuse |  | Reduced | Reduced |  |  |  |  |  |
| **Potash et al., 2000** | Johns Hopkins U. School of Medicine, Maryland, USA | N=251, BD I | Alcoholism |  |  |  |  |  |  | Increased OR=2.6 |  |
| **Cassidy et al., 2001** | John Umstead Hospital, North Carolina, USA | N=392, BD I, inpatients | SUD |  |  |  |  |  |  |  | Increased |
| **Lopez et al., 2001** | Hospital Santiago Apóstol, Vitoria, Spain | N=169, BD I | Alc./drug abuse |  |  |  |  |  |  | Increased with drugs, not alcohol  OR=2.9 (1.0-7.7) |  |
| **Salloum et al., 2001** | WPIC, U. of Pittsburgh, USA | N=126, BD I+II | Alcohol dependence |  |  |  |  |  |  | Trend towards increase in females |  |
| **Salloum et al., 2002** | WPIC, U. of Pittsburgh, USA | N=256, BD I, inpatients | Current alcohol misuse |  |  |  |  |  | Reduced  OR 1.56 (1.01-2.39) | No effect |  |
| **Dalton et al., 2003** | CAMH, U. of Toronto (genetic study), Canada | N=336, BD I+II and schizoaffective disorder | SUD |  |  |  |  |  |  | Increased with drugs, not alcohol, OR=2.09 (1.03-4.21) |  |
| **MacKinnon et al., 2003** | NIMH BD Genetics Initiative, USA | N=603, BD I+II and schizoaffecttive disorder | SUD |  |  |  |  |  |  | No effect |  |
| **Comtois et al., 2004** | U. of Washington, USA | N=1938 (total N=7819),BD, inpatients | Current subst. use problems (predef. criteria) |  |  |  |  |  |  | Increased ORs (for different measures) =1.4-5.3 |  |
| **Verduin et al., 2005** | VA Cooperative Study, 11 sites in USA | N=328, , BD, inpatients |  | No effect | Increased (higher employability) |  |  | Reduced | No effect | No effect |  |
| **Swann et al., 2005** | U. of Texas Mental Sciences Institute, USA | N=48, BD I | Substance abuse |  |  |  |  |  |  | Increased with alcohol, not other substances |  |
| **Singh et al., 2005** | Chandigarh, India | N=80 BD (total N=160) | Substance dependence |  | Increased (fewer unemployed) |  | Reduced |  |  |  | Increased |
| **Weiss et al., 2005** | STEP-BD, USA | N=1000, BD I+II | SUD  current, lifetime | Reduced in current SUD only |  | Reduced | Reduced |  |  | Increased |  |
| **Goldstein et al., 2005** | NIMHs “COBY”, USA | N=446, BD I+II and NOS, children and adolescents | SUD |  |  |  |  |  |  | Increased OR=2.76 (1.21-6.28) |  |
| **Goldstein et al., 2008** | NIMHs “COBY”, USA | N=249, BD I+II and NOS, adoloescents | SUD |  |  |  |  |  | No effect | Increased  OR=2.8 (1.3-6.2) |  |
| **Haro et al., 2006** | EMBLEM, 14 European countries | N=3536, BD I | SUD |  | Reduced | No effect |  | Reduced |  | Increased | Increased |
| **Van Rossum et al., 2008** | EMBLEM, 14 European contries | N=3425, BD I, 12 weeks prospective | SUD |  | Reduced OR=2.47 (1.44-4.26) | No effect | Reduced |  |  |  |  |
| **Manwani et al., 2007** | McLean Hospital, (several treatment programs), Massachusettes, USA | N=115, BD I+II, inpatients, outpatients | SUD |  |  |  |  | Reduced |  |  |  |
| **Baldessarini et al., 2008** | Adelphi Bipolar Disorders Disease-Specific Program, UK and USA | N=429, BD I+II and NOS | AUD |  |  |  |  | Reduced  RR=2.26 |  |  |  |
| **Goldstein and Levitt, 2008** | NESARC, USA | N=1411, BD I | SUD |  |  | No effect (reduced “role emotional” in females |  |  |  |  | No effect |
| **Cardoso et al., 2008** | Hospital de Clinicas de Porto Alegre, Brazil | N=186, BD I+II | AUD | Reduced |  |  | No effect |  | Reduced | Increased | No effect |
| **Sentissi et al., 2008** | INSERM, Paris, France | N=73, BD I+II | SUD |  |  | No effect | No effect |  |  |  |  |
| **Khalsa et al., 2008** | McLean/Harvard First Episode project, Massachusettes, USA. | N=216, BD I | SUD |  |  |  |  |  |  | No effect |  |
| **Baca-Garcia et al., 2009** | NYSPI and Columbia U., New York, USA | N=97, BD | AUD |  |  |  |  | No effect |  | No effect |  |
| **Sajatovic et al., 2009** | Case Western Reserve U. School of Medicine, Ohio, USA | N=113, BD I | ASI-score |  |  |  |  | Reduced |  |  |  |
| **Hoblyn et al., 2009** | Veterans Affairs adm.database, California, USA | N=2963, BDI, II, NOS | SUD |  |  |  |  |  |  |  | Increased for all substances, RR=2.49-3.58 (2.02-5.03) |
| **Mazza et al., 2009** | Catholic U. of sacred heart, Rome, Italy | N=131, BDI+II, cycloth., 1 year prospective | SUD | No effect |  | Reduced | No effect |  | No effect |  |  |
| **Sublette et al., 2009** | NYSPI, New York and WPIC, Pittsburgh, USA | N=138, BDI+II | AUD, SUD |  |  |  |  |  |  | Increased in BD I, OR=2.54 (0.78-8.23)(alc.), OR=4.70 (1.20-18.32) (drugs) and reduced in BD II, OR=0.71 (0.19-2.76 (alc.), OR=0.58 (0.15-2.24) (drugs) |  |
| **Neves et al., 2009** | U. Federal de Minas Gerais, Brazil | N=239, BDI+II, In- and outpatients. | Alcoholism, drug addiction |  |  |  |  |  |  | No effect (increased in bivariate but not multivariate analyses |  |

*Abbreviations: U. = university, SUD = substance use disorder, AUD = alcohol use disorder, OR = odds ratio, RR = risk ratio, HR = hazard ratio.*

*When no specification on type of BD, in/outpatient status etc. is listed, no information was presented in the paper. Substance use disorder is “lifetime” when no information on whether it is current or lifetime is listed. The studies are presented chronologically, except when all or parts of participants are shared between studies. In this case, the studies are grouped together. Effect sizes (OR, RR and HR) are listed with 95% CI in parentheses if these are reported in the study.*

**Part II: *Reported effects of substance use disorders on measures of illness course and clinical characteristics specific to bipolar disorder.***

| **Publication** | **Study** | **Methods and sample charcteristics** | **Type and measure of SUD** | **Illness Course and Clincal Characteristics Bipolar Disorder** | | | | | | | |
| --- | --- | --- | --- | --- | --- | --- | --- | --- | --- | --- | --- |
|  |  |  |  | **Early AAO of BD** | **Recovery** | **Relapse** | **No. of affective episodes** | **Severity of symptoms** | **Mixed episodes** | **Psychosis** | **Rapid cycling** |
| **Morrison et al., 1974** | VA Hospital, San Diego, California, USA | N=38, BD I | Alcoholism | Increased |  |  | No effect |  |  |  |  |
| **Himmelhoch et al., 1976** | Affective Disorder Clinic, Yale U. Medical Center, USA | N=84, manic-depressive outpatients, 5 year prospective | Alcohol and drug abuse |  | Reduced (increased risk of ”poor response” as defined as either lack of remission or relapse during follow-up) | |  |  | Increased |  |  |
| **Keller et al., 1986** | NIMH Collaborative Study on the psychobiology of depression, USA | N=155, BD I, 18 month prospective | AUD |  | Reduced (sign. level not presented) |  |  |  | Increased (sign. level not presented) |  |  |
| **Winokur et al., 1995** | NIMH Collaborative Study on the psychobiology of depression, USA | N=231, BD I+II and schizoaffective disorder, 5 year prospective | AUD |  | No effect | No effect | No effect |  |  | No effect |  |
| **Coryell et al., 1998** | NIMH Collaborative Study on the psychobiology of depression, USA | N=113, 15 year prospective | Alcoholism |  | Reduced |  |  |  |  |  |  |
| **Tohen et al., 1990** | McLean/Harvard First Episode project, Massachusettes, USA. | N=24, BD I, 4 year prospective | Alcoholism |  |  | Increased HR=13.9 (1.4-138.0) |  |  |  |  |  |
| **Tohen et al., 1990** | McLean Hospital, Massachusettes, USA. | N=75 BD I, 4 year prospective | Alcoholism |  |  | Increased HR=8.9 (1.2-12.6) |  |  |  |  |  |
| **Tohen et al., 1996** | McLean/Harvard First Episode project, Massachusettes, USA. | N=123, BD I, 2 year prospective | Current SUD |  | Reduced |  |  |  | No effect |  |  |
| **Sonne et al. 1994** | Medical U. of South Carolina, USA | N=44, BD I+II, in- and outpatients | SUD current/lifetime | Increased |  |  |  | No effect | Increased (dysphoric mania) |  | Increased (mood-swings, sign. level not presented) |
| **Tsai et al., 1997** | Taiwan (Chinese patients) | N=158 BD I+II | AUD | No effect |  |  |  |  |  | No effect (in first episode) | Increased |
| **Keck et al., 1998** | U. of Cincinnati Hospital, USA. | N=134, BD I, inpatients, 1 year prospective | SUD |  | No effect |  |  |  |  |  |  |
| **Pini et al., 1999** | Pisa Center, Italy | N=125, psychotic BD, inpatients | SUD | Increased (in patients with comorbidity in addition to SUD) |  |  |  |  |  |  |  |
| **Goldberg et al., 1999** | New York Hospital, USA | N=204, BD I, inpatients | SUDs | No effect | Reduced for both alcohol or marihuana |  |  | No effect | Increased | No effect |  |
| **Cassidy et al., 2001** | John Umstead Hospital, North Carolina, USA | N=392, BD I, inpatients | SUD | No effect |  |  |  |  | No effect in current mania |  |  |
| **Salloum et al., 2001** | WPIC, U. of Pittsburgh, USA | N=126, BD I+II | Alcohol dependence |  |  |  |  | Increased depr. symptoms in females only |  |  |  |
| **Salloum et al., 2002** | WPIC, U. of Pittsburgh, USA | N=256, BD I, acute manic inpatients | Current alcohol misuse |  |  |  |  | Increased in current episode OR=1.19 (1.05-1.36) | Increased mood lability in current episode OR=3.06 (1.45-6.48) |  |  |
| **Judd et al., 2002** | 5 academic centers, USA | N=146 BDI, 12 years prospective | RDC drug and alcohol use disorders |  | Reduced for drugs, not alcohol |  |  |  |  |  |  |
| **Dalton et al., 2003** | CAMH, U. of Toronto (genetic study), Canada | N=336, BD I+II, and schizoaffective disorder | SUD | Increased |  |  |  |  |  |  |  |
| **Carter et al. 2003** | CAMH, U. of Toronto (genetic study), Canada | N= 320, BD I+II | SUDs | Increased for drugs, not alcohol |  |  |  |  |  |  |  |
| **MacKinnon et al., 2003** | NIMH BD Genetics Initiative, USA | N=603, BD I+II and schizoaffecttive disorder | SUD |  |  |  |  |  |  |  | Increased  OR=1.63 (1.18-2.27) |
| **Nolen et al., 2004** | Stanley Foundation Bipolar Network Site, Utrecht, The Netherlands | N=258, BD I+II, NOS and schizoaffective | SUD |  |  |  | No effect | Increased for mania, not depression |  |  |  |
| **Ernst and Goldberg, 2004** | Payne Whitney Clinic of NY Presbyterian Hospital, USA | N=56, BD I+II, NOS | SUD | Increased  OR=7.71 (1.86-31.94) |  |  |  |  |  |  | Increased |
| **Weiss et al., 2005** | STEP-BD, USA | N=1000, BD I+II | SUD current, lifetime |  | Reduced |  |  |  |  |  |  |
| **Schneck et al., 2004** | STEP-BD, USA | N=456, BD I+II | Substance abuse |  |  |  |  |  |  |  | No effect |
| **Perlis et al., 2006** | STEP-BD, USA | N=1469, BD I+II, 2 years prospective | SUD current, lifetime |  |  | Increased for mania with current SUD, HR=1.61 (1.01-2.57) |  |  |  |  |  |
| **Verduin et al., 2005** | VA Cooperative Study, 11 sites in USA | N=328, inpatients, veterans, public sector | SUD | No effect | Reduced |  | No effect (prior year) |  |  | No effect (current psychosis) | No effect |
| **McKowen et al., 2005** | UCLA Mood Disorders Research Program, USA | N=30, BD I+II | AUD |  |  |  |  |  |  |  | Increaed (with high no. of drinks per day) |
| **Goldstein et al., 2008** | NIMHs “COBY”, USA | N=249, BD I+II, NOS, adoloescents | SUD | No effect |  |  |  | No effect | No effect | No effect |  |
| **Haro et al., 2006** | EMBLEM, 14 European countries | N=3536, BD I | SUD | Increased |  |  |  |  |  |  | No effect |
| **Van Rossum et al., 2008** | EMBLEM, 14 European countries | N=3425, BD I, 12 weeks prospective | SUD |  | Reduced |  |  | Increased for mania, not depression |  |  |  |
| **Goldstein and Levitt, 2008** | NESARC, USA | N=1411, BD I | SUD |  |  |  | No effect (12 month prevalence) |  | Increased in females only |  |  |
| **Cardoso et al., 2008** | Hospital de Clinicas de Porto Alegre, Brazil | N=186, BD I+II | AUD | Increased |  |  |  | Increased for depression, not mania |  | Increased (in first episode) |  |
| **Sentissi et al., 2008** | INSERM, Paris, France | N=73, BD I+II | SUD | No effect |  |  |  |  |  |  |  |
| **Baca-Garcia et al., 2009** | NYS Psychiatric Institute and Columbia U., New York, USA | N=97, BD | AUD |  |  |  |  | No effect |  |  |  |
| **Mazza et al., 2009** | Catholic U. of sacred heart, Rome, Italy | N=131, BDI+II and cyclothymia, 1 year prospective | SUD |  |  | No effect |  | No effect |  |  |  |

*Abbreviations: U. = university, SUD = substance use disorder, AUD = alcohol use disorder, OR = odds ratio, RR = risk ratio, HR = hazard ratio.*

*When no specification on type of BD, in/outpatient status etc. is listed, no information was presented in the paper. Substance use disorder is “lifetime” when no information on whether it is current or lifetime is listed. The studies are presented chronologically, except when all or parts of participants are shared between studies. In this case, the studies are grouped together. Effect sizes (OR, RR and HR) are listed with 95% CI in parentheses if these are reported in the study*
